# Effects of omega-3 supplementation as an adjunct to non-surgical periodontal therapy on periodontal parameters in periodontitis patients: a randomized clinical trial

**DOI:** 10.1186/s12903-022-02569-5

**Published:** 2022-11-21

**Authors:** Fahimeh Rashidi Maybodi, Mahsa Fakhari, Fatemeh Tavakoli

**Affiliations:** 1 Department of Periodontics, Faculty of Dentistry, Shahid Sadoughi University of Medical Sciences, Yazd, Iran; 2 Department of Toxicology and Pharmacology, School of Pharmacy, Shahid Sadoughi University of Medical Sciences, Yazd, Iran

**Keywords:** Fatty acids, Omega-3, Periodontal Diseases, Periodontitis

## Abstract

**Objectives:**

This study aimed to assess the effects of omega-3 fatty acid supplementation as an adjunct to non-surgical periodontal therapy in patients with periodontitis.

**Materials and methods:**

This randomized clinical trial was conducted on 30 patients with periodontitis. All patients received standard non-surgical periodontal therapy, and were randomly divided into two groups of intervention and control by a table of random numbers (*n* = 15). The intervention group consumed 1000 mg natural fish oil soft-gels daily (300 mg Omega-3 marine triglycerides, 180 mg Eicosapentaenoic acid and 120 mg Docosahexaenoic acid) while the control group used soft-gels contained only some soybean oil for 3 months. Clinical attachment loss (CAL), probing depth (PD), and bleeding index (BI) were recorded at baseline (before the intervention) and after 3 months. The two groups were compared regarding the clinical parameters by t-test (alpha = 0.05).

**Results:**

All three clinical parameters decreased in both groups at 3 months compared with baseline (*P* = 0.001). The improvement in PD and CAL in the intervention group was significantly greater than that in the control group (*P* = 0.001); however, the difference in BI was not significant between the two groups (*P* = 0.283).

**Conclusion:**

Omega-3 supplementation as an adjunct to non-surgical periodontal therapy significantly improved the clinical parameters in periodontitis patients compared to soybean oil supplements.

## Introduction

Periodontitis is a multifactorial inflammatory disease, which can cause periodontal tissue destruction and eventual tooth loss. Several factors can affect the susceptibility to periodontal disease and its progression, such as genetics, systemic diseases, and lifestyle including oral hygiene, smoking, stress, and nutrition [1]. Although presence of bacterial biofilm is an important prerequisite for development of periodontitis, the host immune response is a key factor in disease progression [2]. In susceptible patients, if dysbiotic, the adherent microbial biofilm trigger an inflammatory host response that can damage surrounding tissues, including alveolar bone.

Professional subgingival scaling along with plaque control by patients can significantly improve probing depth (PD), clinical attachment loss (CAL), and bleeding on probing [3]. Scaling and root planning (SRP) is the gold-standard treatment for most patients with periodontitisperiodontitis. Nonetheless, different therapeutic strategies have been proposed over the past years to improve the results of SRP and decrease the need for surgical periodontal interventions in patients with advanced periodontitis [4]. For example, various types of mouthwashes have been proposed as an adjunct to periodontal therapy to reduce gingival or periodontal parameters [5]. Of the available chemical mouthwashes, chlorhexidine is the gold-standard antimicrobial mouthwash [6].

Another very common treatment modality is antibiotics usage but when antibiotics are used in the treatment of periodontal diseases, the literature shows that patients are at a probable risk of allergies, nephritis, hematological problems, digestive problems, nervous system disorders and electrolyte problems.[7].

Host modulation treatments have been recently suggested as an adjunct to SRP, aiming to decrease tissue destruction caused by inflammation. These treatments include topical, local or systemic use of medications as a supplemental method during periodontal therapy [8]. Theoretically, different medications may be considered for host modulation such as non-steroidal anti-inflammatory drugs, tetracyclines, or bisphosphonates; however, they were only used for a limited period of time in previous studies since they had the risk of adverse side effects [9].

It has been reported that a low-carbohydrates diet, rich in omega-3 fatty acids, rich in vitamins C and D, and rich in fiber can significantly reduce gingivitis and periodontal inflammation [10].

Some previous studies also mentioned further improvement of periodontal status by the consumption of dietary supplements containing vitamin D [11], vitamin C [12] and probiotics [13] as an adjunct to SRP. Unsaturated omega-3 fatty acids can also be used as a supplement in many chronic inflammatory diseases with fewer side effects. Their optimal efficacy for reduction of inflammation in periodontal disease has been previously reported [14]. Intake of omega-3 fatty acids is necessary since they are not synthesized in the human body. The ratio of omega 3/omega 6 in the body highly depends on their intake through diet [15]. Omega-3 fatty acids include α-linolenic acid, eicosapentaenoic acid, and docosahexaenoic acid, which are found in abundance in fish oil and walnut oil [16].

Omega-3 fatty acids are merged with the cell membrane phospholipids, and serve as a precursor for lipid mediators to control cell signals, alter gene expression, and regulate inflammatory processes [16, 17], resulting in anti-inflammatory effects. Moreover, the metabolism of omega-3 fatty acids results in production of pro-resolving lipid mediators with anti-inflammatory and immunoregulatory effects, inhibiting the migration of immune cells and production of pro-inflammatory cytokines [18]. Furthermore, eicosapentaenoic acid and docosahexaenoic acid have antibacterial effects and can inhibit the activity of some periopathogenic microorganisms such as *Porphyromonas gingivalis*, *Fusobacterium nucleatum*, and *Prevotella intermedia* [14, 19]. Omega-3 supplements do not have significant complications; however, some patients may be allergic to them [20]. Also, omega-3 supplements may interfere with some anti-coagulants and cause hemorrhage [21].

As mentioned earlier, the beneficial effects of omega-3 fatty acids on periodontal parameters have been previously documented [22, 23]. However, some other studies refuted such effects [24–27]. A recent systematic review could not reach a final conclusion on the advantages of omega-3 supplementation for periodontal therapy due to high heterogeneity in the methodology (such as dosage of supplements or duration of use) or inconsistency in the obtained findings and also high risk of bias [28]. Considering the existing controversy on this topic, this study aimed to assess the effects of omega-3 fatty acid supplementation on periodontal parameters in patients with periodontitis.

## Materials and methods

This study was conducted at the Periodontics Department of School of Dentistry, Yazd University of Medical Sciences between August 2021 and March 2022. The study was approved by the ethics committee of this university (IR.SSU.REC.1400.025) and at 17/07/2021 registered in the Iranian Registry of Clinical Trials (IRCT20151013024509N5).

### Trial design

A parallel-design randomized clinical trial was conducted in which, the intervention group received omega-3 supplementation for 3 months after SRP while the control group received placebo for 3 months after SRP. The results were reported in accordance with the Consolidated Standards of Reporting Trials.

### Participants, eligibility criteria, and settings

The inclusion criteria were periodontitis staged as II to IV and graded as B. [29], (II) age between 30 and 70 years, (III) no cigarette smoking or tobacco use, (IV) no history of periodontal therapy, (V) no history of antibiotic use in the past 3 months, (VI) absence of diseases or conditions that would interfere with wound healing such as diabetes mellitus or coagulation disorders, (VII) absence of oral mucosal inflammatory conditions such as aphthous ulcers or lichen planus, (VIII) no intake of medications affecting the periodontium such as anticonvulsants, calcium channel blockers, or immunosuppressant drugs, no history of seafood allergy (IX) and (X) no pregnancy or nursing.

The exclusion criteria were (I) not showing up for the 1-month or 3-month follow-ups, and (II) no or poor adherence to the instructions regarding omega-3 supplementation, or oral hygiene instructions at the 1-month follow-up.

The sample consisted of 30 patients with periodontitis presenting to the Periodontics Department of School of Dentistry, Yazd University of Medical Sciences for periodontal therapy.

### Informed consent

Informed consent was obtained from the patients to participate in the study and to use the results obtained from the study by the post-graduate student.

### Interventions

Written informed consent was obtained from all patients after they were briefed about the study. When gingival inflammation symptoms such as erythema, edema and tendency to bleeding on probing and the interdental CAL ≥ 3 mm was present and apart from the number of tooth loss the diagnosis was recorded as periodontitis with stage II ( moderate periodontitis) to stage IV (advanced periodontitis with extensive tooth loss) according to 2017 international periodontology workshop. As mentioned before, absence of risk factors for example smoking and systemic conditions such as diabetes which affected grading, were considered as inclusion criteria. So progression was as expected (grade B). No full-mouth PA radiographs available, should be considered as one of the limitations of this study.

The CAL, PD and bleeding index (BI) [30] were measured for each patient and recorded at baseline. Next, all patients received SRP with an ultrasonic scaler (Various 350; NSK, Japan) and a manual universal curette (Gracey; Hu-Friday, Chicago, USA) by a post-graduate student of periodontics. They also received prophylaxis with a prophy brush and prophy paste. The modified Bass tooth brushing technique was then instructed to patients on a model. Patients were asked to brush their teeth twice a day after meals.

Next, the patients were randomly assigned to two groups, and were standardized in terms of age, gender distribution, and severity of periodontal disease based on the mean baseline PD, CAL, and BI.

Fish oil mercury-free soft-gels (*n* = 1350, 1000 mg each) containing natural fish oil ( 300 mg Omega-3 marine triglycerides, 180 mg Eicosapentaenoic acid and 120 mg Docosahexaenoic acid) and control soft-gels containing only soybean oil were produced by Yas- Kavir pharmaceuticals. The intervention group received 1000 mg omega-3 soft-gels while the control group received soft-gels containing very small amount of soybean oil to only make the gelatinous capsule not look empty (150 mg). Both groups were instructed to use one soft-gel daily for 3 months [23]. Accordingly, each group received three bottles, each containing 30 soft-gels. The patients were recalled at 1 month to ensure their adherence to oral hygiene instructions. The patients were requested to continue using the supplement. The patients were recalled again at 3 months, and their PD, CAL, and BI were measured again.

### Outcomes (primary and secondary)

The primary outcomes of this study was evaluating CAL before and after the intervention. The secondary main objective of this study was to assess BI & PD probable changes according to the usage of omega-3 supplementation following SRP on clinical improvement in patients with periodontitis.

### Sample size calculation

The sample size was calculated to be at least 15 in each group according to a study by Kruse et al., [25] considering alpha = 5%, study power of 80%, standard deviation of CAL to be 0.7, and a minimum significant difference of 0.6 unit in CAL between the two groups.

### Interim analyses and stopping guidelines

No interim analyses were performed, and no stopping guidelines were established.

### Randomization

The patients were randomly assigned to the intervention and control groups by a table of random numbers generated by Random Allocation Software version 1.0.( Mahmood Saghaei, Iran).

### Blinding

The study had a double-blind design. Both omega-3 and soybean oil soft-gels had the same shape and color and similar packaging. A pharmacist not involved in the study coded the soft-gels A and B based on their content and administered them among the patients. The dental clinician who measured the clinical periodontal parameters and the patients were not aware of the group allocation of soft-gels. The statistician who analyzed the data was also blinded to the group allocation of data.

### Statistical analysis

Data were analyzed by SPSS version 25 (SPSS Inc., IL, USA). Normal distribution of data was evaluated by the Kolmogorov-Smirnov test. Comparisons were made by paired t-test and t-test at 0.05 level of significance.

## Results

### Participant flow

**Fig. 1:**
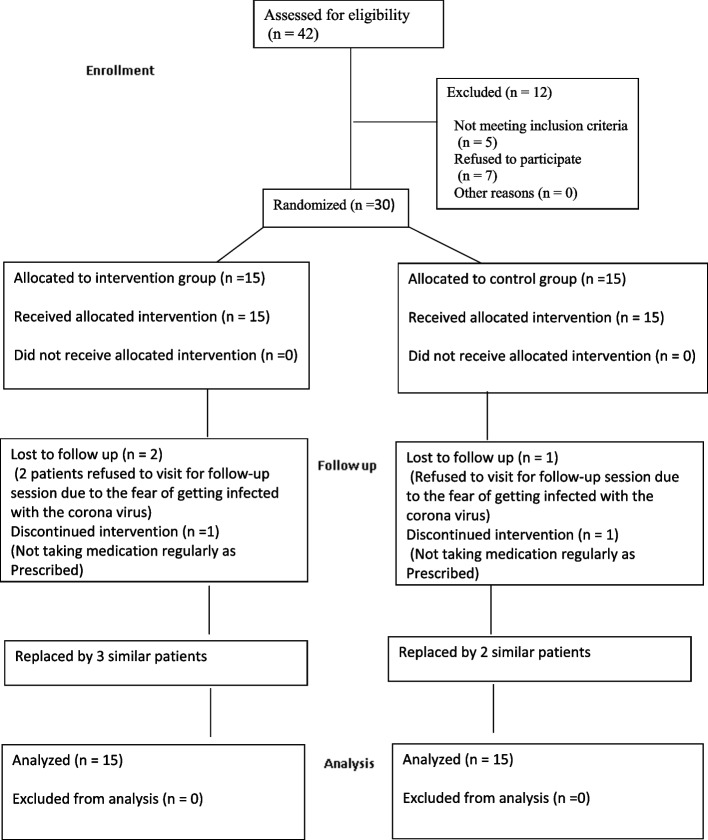
Flow diagram of patient selection

A total of 30 patients in 2 groups of 15 were evaluated in this study. Fig 1 shows the flow-diagram of patient selection. The two groups were standardized in terms of age, gender, and baseline clinical periodontal parameters as shown in Table 1. There were 7 females (46.7%) and 8 males (53.3%) in the intervention group, and 9 females (60%) and 6 males (40%) in the control group. The two groups were not significantly different regarding gender distribution (*P* = 0.358).

**Table 1:** Standardization of the two groups at baseline using t-test (*n* = 15)

Group	Group	Mean	Std. deviation	*P*-value*
Age (years)	Intervention Control	45.467 42.867	7.698 8.416	0.602
Baseline PD (mm)	Intervention	5.067	0.982	0.137
	Control	4.507	1.018	
Baseline CAL (mm)	Intervention Control	5.240 4.667	1.041 1.010	0.137
Baseline BI	Intervention	78.533	9.869	0.789
	Control	79.533	10.432	

*PD* Pocket depth, *CAL* Clinical attachment loss, *BI* Bleeding index,

*t-test at 0.05 level of significance

### Harms

No patients were harmed during the study and no side effect was reported.

### Subgroup analysis

The Kolmogorov-Smirnov test confirmed normal distribution of data (*P* > 0.05). Table 2 presents the mean PD, CAL and BI in the two groups at baseline and at 3 months after the intervention. As shown, all parameters significantly decreased at 3 months compared with baseline in both groups (*P* < 0.05). The intra-group difference of the parameters is mentioned in Table 2.

**Table 2:** Mean PD, CAL and BI and in the two groups at baseline and at 3 months after the intervention

Group	Variable	Time	Mean	Std. deviation	*P-value
Intervention	PD	Baseline	5.067	0.982	0.000
		3 months	2.653	0.843	
		Intra-group difference	2.413	0.8	0.000
	CAL	Baseline	5.24	1.04	0.000
		3 months	3.07	0.61	
		Intra-group difference	2.166	0.745	0.000
	BI	Baseline	78.53	9.86	0.000
		3 months	49.80	16.06	
		Intra-group difference	28.733	14.424	0.000
Control	PD	Baseline	4.507	1.018	0.000
		3 months	3.340	0.940	
		Intra-group difference	1.166	0.654	0.000
	CAL	Baseline	4.66	1.01	0.000
		3 months	3.57	0.92	
		Intra-group difference	1.093	0.681	0.000
	BI	Baseline	79.53	10.43	0.000
		3 months	56.26	15.09	
		Intra-group difference	23.266	12.892	0.000

*Paired t-test at 0.05 level of significance

The improvement in PD and CAL in the intervention group was significantly greater than that in the control group (*P* = 0.001); however, the difference in BI was not significant between the two groups (*P* = 0.283).

## Discussion

This study assessed the effects of omega-3 fatty acid supplementation on periodontal parameters in patients with periodontitis. The sample size of this study was similar to that in a study by Keskiner et al., [26] higher than that in studies by Martinez et al., [25] and Rampally et al., [31] and smaller than that in studies by Stańdo et al., [32] and Shalaby et al. [33]. The dosage of omega-3 administered in the present study was similar to some previous studies [31, 34, 35]. No side effect was reported by patients in the present study; however, Stańdo et al. [32] reported that patients complained of nausea and mouth malodor (fish smell) as side effects. Selection of 1- and 3-month follow-ups was also in line with previous studies [31, 32, 36].

Our results showed that all three clinical parameters decreased in both groups at 3 months compared with baseline; however, the difference in BI was not significant between the two groups. Bleeding on probing has a strong correlation with inflammatory processes due to the presence of dental plaque. Usually, if dental plaque is not removed properly for about one week, the primary clinical signs of inflammation such as erythema and bleeding on probing will appear on probing but the increase in PD and CAL, will take longer time to occur [37]. Since the final assessment was done at 2 months after the first follow-up session, it is possible that patients did not perfectly adhere to oral hygiene instructions for complete plaque removal, resulting in an increase in their BI. Also, it should be noted that patients who willingly participate in a study related to oral hygiene status often take better care of their teeth and better adhere to oral hygiene instructions, as they feel they are under close supervision. This phenomenon is referred to as the Hawthorne effect [38]. Of course it doesn’t seem that there is a guarantee for the continuation of health behaviours with the same quality. Of limited studies that assessed BI, the present results were in agreement with those of El-Sharkawy et al., [39] and Martinez et al. [25]. However, Stańdo et al. [32] reported a significant reduction in bleeding on probing in the intervention group, compared with the control group. They also evaluated patients both at 3 weeks and 3 months intervals. Thus, different between their results and the present findings may be due to the higher dose of omega-3 used in their study (3000 mg), or better adherence of patients to plaque control measures, or the fact that patients also received instructions to use inter-dental brush (which was not instructed in the present study), in addition to toothbrush and dental floss. Of clinical parameters, CAL and PD were evaluated in almost all similar previous studies. Stańdo et al., [32] and many others [34, 36, 39] reported results similar to the present findings regarding the improvement in clinical parameters as the result of omega-3 supplementation. Martinez et al., [25] and Keskiner et al. [26] reported a reduction in CAL and PD in both the intervention and control groups but the difference between the two groups was not significant. In the present study, the difference in CAL and PD between the two groups was > 1 mm, and therefore, it was considered as clinically significant according to the agreement among the administrators of this research.

This study had several strengths. At the onset of study, the patients were evaluated to ensure absence of confounders such as cardiovascular diseases and diabetes mellitus, history of antibiotic therapy in the past 3 months, and pregnancy to ensure no effect of confounders on the results. Martinez et al. [25] did not exclude smokers and Rampally et al. [31] evaluated type II diabetes mellitus patients. Also, the two groups were standardized in terms of the mean age, gender distribution, and severity of periodontal disease in the present study to minimize their possible distorting effect in the periodontal healing process. Both groups received SRP and oral hygiene instructions. Also, the control soft-gels were identical to the omega-3 soft-gels. So the apparent similarity of the intervention was observed in all stages of the study.

This study had some limitations as well. During the COVID-19 pandemic, some patients refused to participate in a dentistry research project or did not show-up for the follow-ups due to the fear of infection risk. Therefore we had to replace these patients with new ones Thus, longer follow-ups could not be scheduled due to the longer sampling and execution phases.

Another limitation of this study was that the omega-3 index was not measured as one of the objectives of the study, but considering the location of the study, which was in Yazd, a desert region in the center of Iran, this index was expected to be not at a desirable level because the consumption of seafood is not common in the residents’ usual diet due to the distance from the sea, as well as the limitation of the production of farmed fish due to the lack of water. Apart from this, the patients referred to the faculty as a governmental center are often from the weaker sections of the society in terms of socio-economics who most likely cannot afford seafood. Calculating this index in future studies is suggested.

It was ideal that a completely neutral placebo was used in the control group, but considering the similar appearance to the capsules used in the intervention group and the safety of the substance and no omega-3 content, we finally reached soybean oil in limited quantities. Further studies are also required with a larger sample size and longer follow-up periods. Also, the efficacy of omega-3 supplementation along with antibiotic therapy or periodontal surgery should be evaluated in further investigations.

## Conclusion

Omega-3 supplementation for 3 months as an adjunct to non-surgical periodontal therapy significantly improved the clinical parameters in periodontitis patients compared to soybean supplementation.

## Data Availability

The datasets generated and/or analyzed during the current study are not publicly available due to privacy and ethical concerns but are available from the corresponding author on reasonable request.

## Abbreviations

CAL: Clinical attachment loss
PD: Probing depth
BI: Bleeding index
